# Structure Driven Prediction of Chromatographic Retention Times: Applications to Pharmaceutical Analysis

**DOI:** 10.3390/ijms22083848

**Published:** 2021-04-08

**Authors:** Roman Szucs, Roland Brown, Claudio Brunelli, James C. Heaton, Jasna Hradski

**Affiliations:** 1 Pfizer R&D UK Limited, Ramsgate Road, Sandwich CT13 9NJ, UK; roland.brown@pfizer.com (R.B.); claudio.brunelli@pfizer.com (C.B.); james.heaton@pfizer.com (J.C.H.); 2 Department of Analytical Chemistry, Faculty of Natural Sciences, Comenius University in Bratislava, Mlynská Dolina CH2, Ilkovičova 6, SK-84215 Bratislava, Slovakia; hradski1@uniba.sk

**Keywords:** Quantitative Structure Retention Relationships, chromatographic method development, pharmaceutical analysis

## Abstract

Pharmaceutical drug development relies heavily on the use of Reversed-Phase Liquid Chromatography methods. These methods are used to characterize active pharmaceutical ingredients and drug products by separating the main component from related substances such as process related impurities or main component degradation products. The results presented here indicate that retention models based on Quantitative Structure Retention Relationships can be used for de-risking methods used in pharmaceutical analysis and for the identification of optimal conditions for separation of known sample constituents from postulated/hypothetical components. The prediction of retention times for hypothetical components in established methods is highly valuable as these compounds are not usually readily available for analysis. Here we discuss the development and optimization of retention models, selection of the most relevant structural molecular descriptors, regression model building and validation. We also present a practical example applied to chromatographic method development and discuss the accuracy of these models on selection of optimal separation parameters.

## 1. Introduction

Pharmaceutical analysis is an important area of chemical analysis used to support diverse and excessively complex activities associated with drug development. The application of Reversed-Phase Liquid Chromatography (RP-LC) is ubiquitous in the support of process chemistry optimisation, formulation development as well as key quality control assessment for the release of materials designated for all stages of pre-clinical and clinical trials.

In process chemistry development, RP-LC is commonly used to assess the assay/purity of starting materials, isolated synthetic intermediates and Active Pharmaceutical Ingredients (APIs). This usually requires baseline separation of all known components of complex mixtures, their identification and subsequent quantitation. This is performed in accordance with the International Council for Harmonisation of Technical Requirements for Pharma-ceuticals for Human Use guidelines as applied to product specification, impurities management and method validation [1,2,3]. In addition, purging of process related impurities, synthetic by-products and key degradants requires their chromatographic monitoring at all relevant interventions (e.g., isolation steps). Process chemistry understanding relies heavily on the application of RP-LC. Chemists are required to understand the impact of synthetic parameters on the quality of their processes which make important starting materials, intermediates and final API. This is an essential requirement of commercial synthetic route development. Lastly, the understanding of degradation also requires chromatographic separation of key degradation products from the main component and their subsequent identification and quantitation [4,5,6].

In order to nominate commercial synthetic process, chemists often generate a relatively large number of hypothetical chemical structures that could be generated as process related impurities. These could be formed as by-products due to impurities present in starting materials or due to side reactions. Another potential for the formation of these undesirable components is degradation reactions which take place either during synthesis or during storage. Realistically, many of these theoretical components will never be observed. However, the analytical methodology (e.g., RP-LC) supporting synthetic process development should be able to at least detect them, if indeed they were to form under certain extreme synthetic or storage conditions.

The satisfactory performance of chromatographic methods can only be guaranteed for a defined/known sample composition. This can be referred to as the key predictive sample set (KPSS), for which the given method was developed and subsequently validated. Evolving requirements during pharmaceutical development, such as changes in synthetic or formulation processes, may lead to alteration of the KPSS, for example in order to manage new process related impurities or degradation products. Examples may include changes in sources of synthetic starting materials, alteration of process chemistry conditions or formulation manufacturing parameters. An inherent, if perhaps somewhat obvious, constraint of chromatography lies in the fact that unless these new KPSS components are physically available, e.g., obtained either by synthesis or purification, it has been virtually impossible to predict whether the current version of chromatographic method used to support particular synthetic or formulation development activities will be able to detect and quantify them. One way to overcome this is to either synthesise these components or to obtain them by purification. The production of compounds whose sole purpose is to de-risk existing analytical methodology, and which may never be formed under “normal” conditions, may be costly and time consuming. Consequently, such activities are often deferred to the latter stages of the drug development lifecycle. Should it transpire, at this stage, that the existing chromatographic method is not capable of detecting these components, should they form, method re-development activity may be triggered. At best, this would necessitate the considerable effort of repeat method robustness and validation work, followed by retest of samples previously analysed using the insufficiently selective methodology. At worst, prior development decisions, for example around synthetic process or formulation, made on the basis of what now proves to be incomplete information, may then need to be revisited.

Chromatographic method development typically starts with the definition of requirements for the capabilities of the analytical technique being used. However, significant consideration is also paid to final product specification. Method performance understanding includes at least following parameters: minimal tolerable resolution of key components and determination of the accuracy, precision and sensitivity or range requirements. Once the necessary performance criteria are understood, and the separation mode capable of achieving these is identified, the next step in the method development process is to select suitable combination of the stationary phase [7], mobile phase (solvent) and pH. This critical step, which ultimately affects the robustness of the method, is in present-day analytical laboratories carried out by combinations of experimental screening [8]. Employment of in-silico prediction tools, capable of calculating key physicochemical properties (e.g., logP, pKa, aqueous/solvent solubilities) of pharmaceutical substances are employed to assist in method design decision making. One example of such software is ACD/Labs Percepta (ACD/Labs, Toronto, ON, Canada). Selection of suitable stationary and mobile phases is followed by more detailed optimisation, for example the column temperature, content of the organic solvent in isocratic or gradient elution, pH of the mobile phase and concentration of the buffer and/or ion pairing reagent. Such optimisation can be carried using an “One Factor at a Time” approach. However, modern approaches employ multi-factorial interpolation software such as ACD/Labs LC Simulator (ACD/Labs, Toronto, ON, Canada) or DryLab (Molnar-Institute, Berlin, Gemany). These enable extrapolation of a relatively small number of experiments that lead to accurate prediction of chromatographic retention times within the intervals of tested conditions.

Alternative approach to de-risking chromatographic methods, and which does not rely on the availability of all hypothetical components of KPSS, is to build sufficiently accurate regression retention models. These are often referred to as Quantitative Structure Retention Relationship (QSRR) models, which can be used to predict retention of these new KPSS components. In QSRR models a mathematical relationship is built between molecular descriptors (or features) and measured retention times, factors or indexes. If relevant structural descriptors can be obtained for a given hypothetical structure, then its retention time can be predicted using the QSRR model. This facilitates assessment of the separation method (de-risking) for potential co-elutions with other sample components. Although the concept of QSRR is not new [8,9,10,11,12], the last decade brought significant expansion of its application especially in the pharmaceutical industry. The renewed interest in this field is probably triggered by progress in availability of diverse structural descriptors [13,14,15,16,17,18], structure geometry optimisation software as well as broad availability of feature selection and regression algorithms [19]. Progress in high performance computing, as well as more widespread and affordable access to it, has inevitably played a significant role in this development. In liquid chromatography, which is by far most frequently used technique in pharmaceutical development, QSRR models were developed for RP-LC, Hydrophilic Interaction Liquid Chromatography and Ion Chromatography separation modes, with applications ranging from method development to non-targeted screening for metabolomics [9], environmental or food pollutants and toxins. These applications, published between 2015 and 2020, were recently extensively reviewed [20]. Analytes for which QSRR models were built range from small molecules, lipids [20] to peptides and proteins [21,22,23,24,25].

In addition to the de-risking of analytical methods, further benefits of QSRR models can be derived from their ability to provide complimentary information in support of structural elucidation challenges. This is a common challenge to both the pharmaceutical industry and in metabolite identification in metabonomic studies [9]. For example, in situations where multiple structural hypotheses remain consistent with available spectroscopic (e.g., Nuclear Magnetic Resonance or Mass Spectrometry) data, additional information based on retention time matching with QSRR prediction might usefully narrow the field. Even if not viewed as definitive, such information might certainly help drive business decision making, for example prioritising which proposed chemical entity should be synthesized first in order to confirm the identity of unknown chromatographic peaks observed in samples.

In this contribution, we describe how the development of accurate retention models can be used to de-risk chromatographic methods in instances where a previously unseen component is postulated. We will describe an optimized approach to model development which includes selection of molecular descriptors using feature algorithms. Model validation, as well as practical application of these models to predict retention extrapolated from a small number of experiments, will also be discussed.

## 2. Results and Discussion

### 2.1. Development of the Statistical Retention Models

As stated previously, the objective of the development of statistical retention models is to create a mathematical relationship between measured retention time and chemical structure. The process of building QSRR models typically starts with data collection. The purpose of this is to create a database of chemical structures and corresponding retention times. A recent review [20] lists number of data sources that have been used to build QSRR models. The numbers of compounds in these databases vary from few tens of compounds to several hundreds or even thousands. Although the perception that the larger datasets typically generate more accurate predictions prevails among researchers, this perception was successfully challenged in some recent publications, in which it was demonstrated that significantly smaller datasets of compounds bearing structural similarity to the analytes of interest generated highly accurate models [26,27,28]. A certain degree of variability of retention times can occur if measured at different time points or utilising different batches of stationary phases. It is therefore preferential to generate the entire dataset in as few chromatographic injections as possible. However, if additional data is required for the purpose of creating QSRR models it is essential that it is collected in a well-controlled environment.

Chemical structures need to be converted into their numerical representation by expressing them through structural descriptors. Structural descriptors can range from measured or calculated physicochemical properties such as octanol-water partition coefficients, to a series of theoretical descriptors which are products of complex cheminformatics algorithms [29]. Contemporary software packages can generate large numbers of structural descriptors (features). It is usually necessary to apply some form of data pre-processing to eliminate constant or nearly constant features as well as those which are highly correlated. Finally, when large numbers of descriptors are generated, an evolutionary searching or genetic algorithm is required to identify or preserve those which positively impact model performance [30]. Selection of suitable regression algorithms can also have a major impact on the accuracy of predictions. There is a relatively large number of classification and regression algorithms available in commercial or open source platforms e.g., WEKA [19,31]. Selection and optimisation of these algorithms can be carried out either manually or by automated procedures [32]. The final step in QSRR model development is usually model validation, which provides an estimate of how accurate the prediction of retention time might be for a hypothetical chemical entity.

#### 2.1.1. Data Collection, Molecular Descriptor Calculation and Data Preprocessing

**Figure 1:**
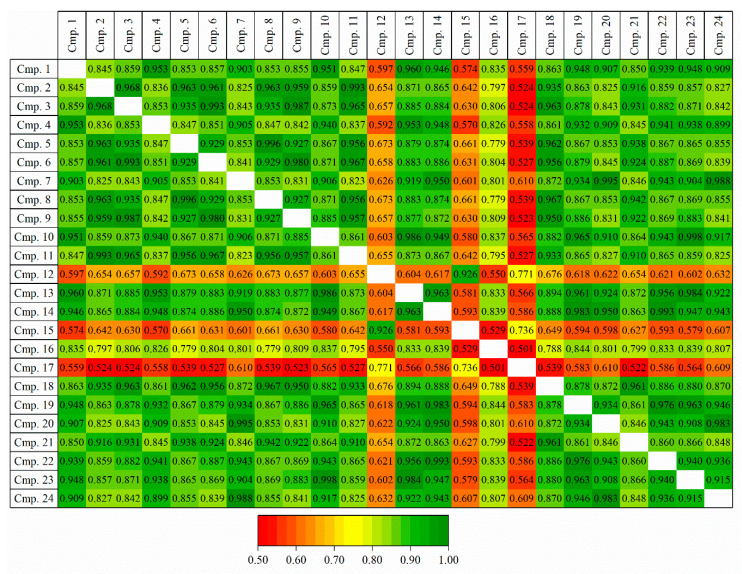
Pairwise structural similarities expressed as Tanimoto index. See text for details.

API and 23 related components from a representative API development program, were selected for initial screening in which multiple stationary and mobile phases were tested for overall best chromatographic performance (see Materials and Methods for details). The mixture of 24 components, being comprised of API, synthetic intermediates, process related impurities, synthetic by-products and degradation products, from the same development program exhibited high degree of structural similarity. Figure 1 shows pairwise structural similarities expressed as Tanimoto index [33] which was calculated using ACD/Labs Spectrus DB (ACD/Labs, Toronto, ON, Canada). Nearly 75% of pairs have similarities higher than 0.8, only three compounds exhibit lower pairwise similarities in the range 0.5–0.7. The high similarities within the dataset are in line with previously published papers in which a relatively small numbers of structurally similar compounds were used to build accurate QSRR models [26,27,28]. For each geometry-optimised 3-D molecular structure, three types of (native) descriptors were calculated, Dragon (4886 descriptors), MOE (256 descriptors) and VolSurf+3D (128 descriptors) (see e.g., [29] for details of what descriptors are and how are they calculated). All zero variance and highly correlated (Correlation Coefficient (R) > |0.85|) descriptors were eliminated. Of multiple highly correlated descriptors, the one with the best correlation with the retention time was preserved [34,35].

#### 2.1.2. Generation of Training and Test Sets

From the entire dataset of 24 compounds, 3 random components were removed, and these were used as the external test set. The remaining 21 compounds formed the training set that was used to identify significant descriptors and to build and optimize regression models. This process was repeated 8 times, every time removing different 3 components. This way 8 training sets /test set combinations were created. The superscripts T1 to T8 in Tables S1 and S2 indicate which training set/test set combination compounds belong to.

#### 2.1.3. Selection of Molecular Descriptors

Evolutionary search (ES) algorithm combined with Multiple Linear Regression (MLR) implemented in Weka [31] was was used to select significant descriptors. One thousand generations were calculated with a population size of 100. The mutation probability was set to 2% and the cross-over probability was set to 6%. Because of the random nature of evolutionary searching, this selection was applied to every training set and repeated three times for all native descriptors (Dragon, VolSurf+3D and MOE) as well as all combinations of descriptors (Dragon & VolSurf+3D, Dragon & MOE, VolSurf+3D & MOE, Dragon & VolSurf+3D & MOE). Root Mean Square Error (RMSE) calculated from 7-fold cross validation applied to each training set was used to identify and select significant descriptors. Thirty descriptors most frequently selected by ES are listed in Table 1.

#### 2.1.4. Selection of Regression Algorithm

**Figure 2:**
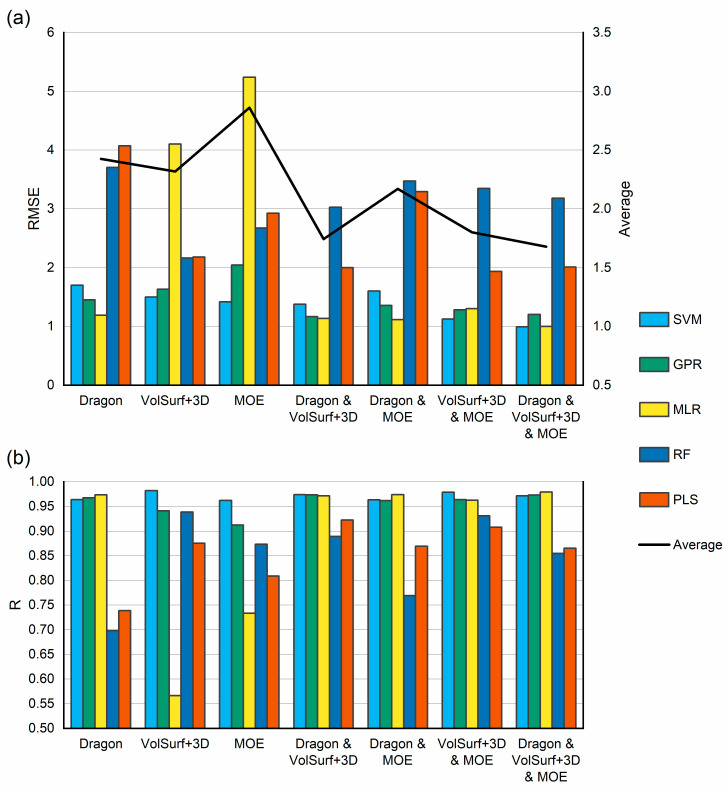
Comparison of Root Mean Square Error (RMSE) (**a**) and Correlation Coefficient (R) (**b**). For all calculated descriptors, their combinations and all regression algorithms. Each bar corresponds to the average value for all training sets. Figure 2a also contains average value for all applied algorithms. SVM: Support vector machine; GPR: Gaussian processes regression; MLR: multiple linear regression; RF: random forest; PLS: partial least squares.

Five regression algorithms (Table 2) implemented in WEKA [31] were applied to all training sets. Each training set consisted of either native descriptors, or their combinations, and were selected by ES as described above. For each training set RMSE as well as R were calculated using 7-fold cross validation. Results are summarized in the Table S3. Figure 2 shows the RMSE (a) and R (b) averaged for all 8 training sets. In addition, Figure 2a also shows the average RMSE values for all applied regression algorithms. It can be seen from Figure 2, that mixed descriptors provide marginally better performance than native descriptors and that Support Vector Machine (SVM) and Gaussian Processes (GPR) regression algorithms consistently outperform MLR, Random Forest (RF) and Partial Least Squares (PLS). Overall best performance was obtained using a mixture of all descriptors and the SVM algorithm. Further attempts to optimise the SVM hyperparameters, such as the complexity factor for example, as well as the exponent in the Normalized Polynomial Kernel did not lead to further improvement of RMSE or R values. Therefore, it was decided to use the WEKA default values i.e., complexity factor was set to 1.0 and the exponent 2.0. The best performing algorithm and the mixture of all 3 descriptors were used to validate the model.

#### 2.1.5. Model Validation

**Figure 3:**
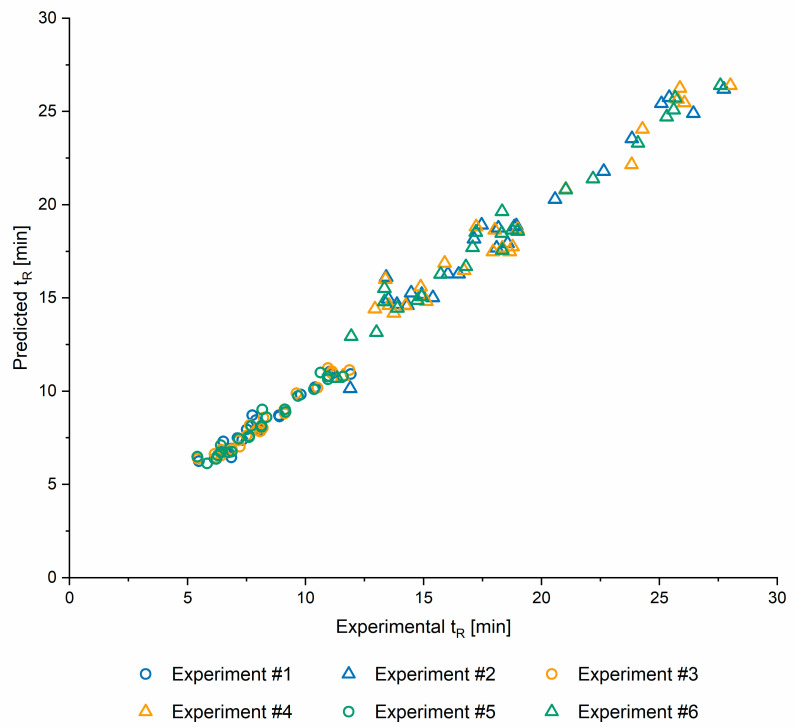
Predicted vs experimental retention times (t_R_) for 6 screening conditions. See Table 4 for the details of experiments.

In order to assess the ability of QSRR models to predict retention times of compounds that were not used in their development or optimisation, retention times for eight test sets, created as described in the Section 2.1.2, were predicted. This was repeated for all six screening conditions as described in the Section 2.2. QSRR predicted retention times are shown in the Table S1 and Figure 3 demonstrates the match between QSRR predicted and experimentally determined retention times. Finally, the corresponding RMSE and R values are provided in the Table 3.

### 2.2. Application to Method Development

**Figure 4:**
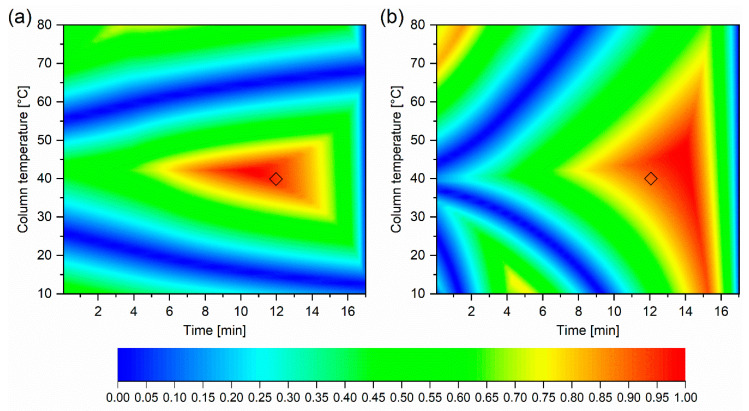
Resolution heat map for key predictive sample set (KPSS). Intensity represents overall chromatogram resolution. High resolution is depicted by red color, low resolution is depicted by blue color. (**a**) constructed from experimental retention times. (**b**) constructed from Quantitative Structure Retention Relationship (QSRR) predicted retention times. The diamond indicates the center point selected from the model created from experimental retention times.

**Figure 5:**
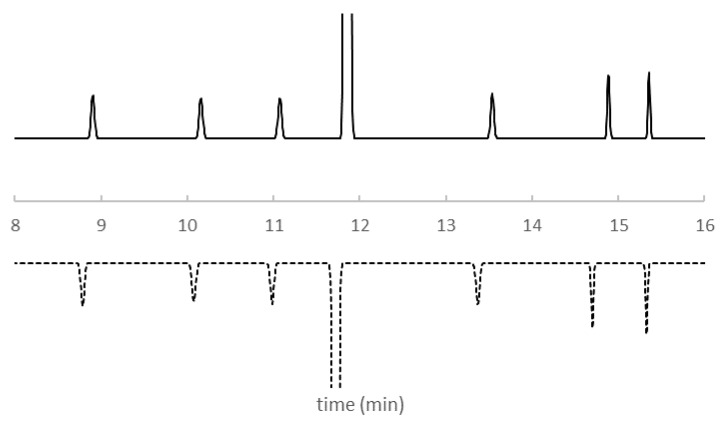
Predicted chromatogram for KPSS components from the retention model built from experimentally determined retention times (RtModel_EXP_) (solid line) and and the retention model built from QSSR predicted retention times (RtModel_QSRR_) (dashed line). Column temperature 40°C. Gradient profile: Time = 0 min, %B = 15%; Time = 12 min, %B = 45%; Time = 17 min, %B = 95%. See Materials and Methods for other details.

As described in the introduction, optimisation is performed once a suitable stationary and mobile phase, buffer, and pH [20] is selected. At this stage, it is typically column temperature and the content of organic modifier in the mobile phase (Gradient time = tG [min]) that are optimised. The details of the initial six experiments are presented in Table 4. Experimental retention times for KPSS for these experiments are shown in Table S2. These measured retention times were extrapolated using the ACD/Labs LC Simulator software. Linear extrapolation was used for the mathematical relationship between natural logarithm of the retention factor of each component (lnk) in the KPPS and tG [min]. Quadratic extrapolation was applied to the relationship between lnk and 1/T where T [Kelvin] is the column temperature. Figure 4a shows the resolution of every component of the KPSS for all combinations of tG and 1/T. For the purposes of clarity, the retention model built from experimentally determined retention times is depicted as RtModel_EXP_. The optimal temperature and gradient composition, the so-called centre point, were selected to consider maximum method robustness i.e., where the overall resolution is maximum and least affected by alteration of T or tG (Figure 4). Figure 5 shows the separation at the optimal temperature and gradient.

In order to assess the suitability of the QSRR, we have essentially replicated the process described except that in this case, instead of measured retention times, we used QSRR predicted retention times (Table S1) as described above (see Section 2.1). Again, for the purpose of clarity this retention model is depicted as RtModel_QSRR_. Agreement between predicted chromatographic separation of KPSS components from RtModel_EXP_ and RtModel_QSRR_, at the experimental conditions corresponding to the centre point is demonstrated in Figure 5. For this subset of compounds, the retention times predicted from RtModel_EXP_ and RtModel_QSRR_ are nearly identical. Also, the resolution heatmap constructed from QSRR predicted retention times, although not entirely identical to the one constructed from experimentally obtained retention times, indicates similar optimal resolution of all compounds belonging to KPSS (Figure 4b). This may not be the case for all compounds as the accuracy of prediction varies from compound to compound. This is also demonstrated in the Figure 3.

**Figure 6:**
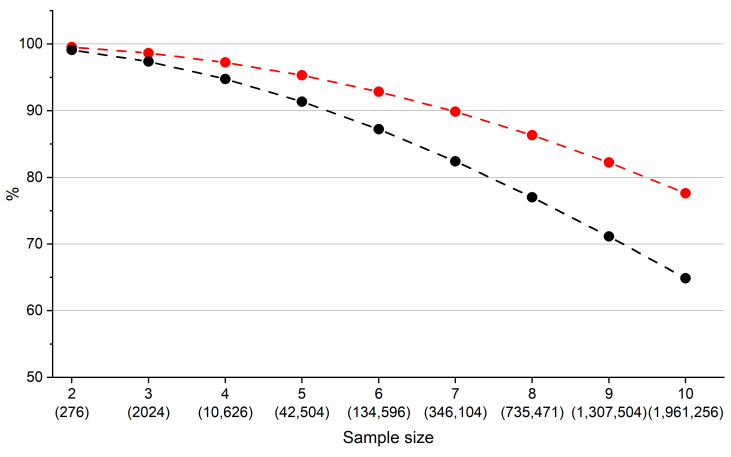
Portion (%) of all combinations of compounds containing two to ten components for which RtModel_EXP_ and RtModel_QSRR_ predicted baseline separation (Resolution Coefficient (RC) = 1). The total number of combinations evaluated is in parentheses. Black line corresponds to model built from predicted data and red line corresponds to model built from mixture of predicted and experimental data. See text for details.

In order to compare retention times predicted from RtModel_EXP_ and those predicted from RtModel_QSRR_ we used all 24 compounds. We then created all possible combinations of two to ten components from this compound set. For each of these combinations we calculated a resolution coefficient (*RC*) according to Equation (1):

(1) RC= ∏i,j1e(RslimitRsi,j−1)

where *Rs_limit_* = 1.25 is minimal satisfactory resolution between two components and *Rs_i,j_* is the actual chromatographic resolution between two components in the mixture. If the *Rs_i,j_* is equal to or exceeds *Rs_limit_* then it is set to *Rs_limit_*. The RC indicates that if the resolution between two components is equal to or exceeds *Rs_limit_* then the RC has a value of one. Whereas, if the resolution between two components is zero then the RC value will also be zero (i.e., 1/*e*^∞^ ≈ 0). Therefore, all other values will fall between values of zero and one. Note that for the calculation of the resolution between two components we used average peak width of 0.1 min. The black line in Figure 6 shows the portion of all combinations for which both models (RtModel_EXP_ and RtModel_QSRR_), predicted baseline separation of all components in the mixture (*RC* = 1).

This data demonstrates that of all theoretical mixtures containing up to seven components which were separated with a resolution of at least 1.25, more than 80% were identified with both models. Even for the most complex mixtures containing ten components, nearly 65% of all combinations were identified with both models. It can be concluded that once QSRR derived retention times are established they can be used to identify conditions in which all components are fully separated. However, the observation described in Figure 6 (black line) represents an extreme case since we are comparing a model built from entirely experimental data with one built from entirely QSRR predicted data. Practically, this scenario will almost always be applied to a mixture of components, for some of which the measured data will be available. We simulated this scenario by randomly replacing approximately 20% (5 out of 24) of retention times obtained from RtModel_QSRR_ with retention times obtained from RtModel_EXP_. As shown in Figure 6 (red line), there were noticeable increases in the proportion of mixtures identified as baseline separated in both models. In practical terms, we usually have many experimentally determined retention times available and few QSRR determined data. We would typically be looking at 2–5 components with which to estimate successful separation. These components are likely to be subtle molecular modifications within the acceptable structural similarity properties of the model.

**Figure 7:**
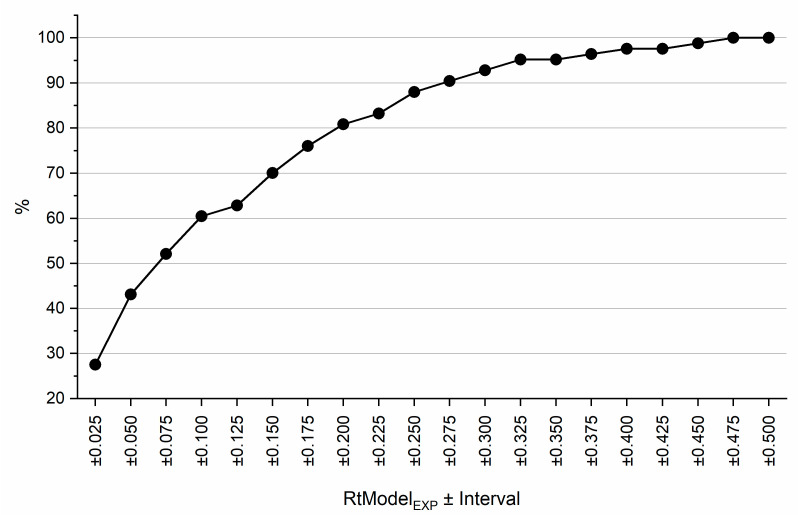
P. Portion (%) of pairwise RC values calculated from RtModel_QSRR_ falling within certain interval RC values calculated from RtModel_EXP_. See text for details.

Lastly, pairwise resolutions were calculated for all 24 compounds determined using both QSRR and experimentally determined retention times. The same assumptions regarding the peak widths as in previous calculations were made. All pairs that exhibited resolution higher than 20 were excluded as these components would always be separated even if the error of prediction was excessive. RC values for all remaining pairs were calculated for retention times predicted from RtModel_EXP_ and RtModel_QSRR_. RC values for these models were compared. Figure 7 shows what proportion of pairwise RC values calculated from RtModel_QSRR_ which falls within specified intervals of RC values calculated from RtModel_EXP_. This figure demonstrates that in excess of 60% of pairwise RC values obtained from RtModel_QSRR_ fall within ±0.1 of RC values obtained from RtModel_EXP_. This again indicates that likelihood of making correct decision with regards to selection optimal separation conditions based on QSRR derived models is high.

## 3. Materials and Methods

### 3.1. Instrumentation

All experiments were performed using an Agilent 1290 – Infinity UHPLC (Agilent Technologies, Waldbronn, Germany) liquid chromatography apparatus equipped with a diode array detector, autosampler, and thermostat. Quadrupole time-of-flight mass spectrometer Agilent 6550i (Agilent Technologies, Singapore) was employed to track chromatographic peaks between different methods. Chromatographic data were collected and processed using a MassHunter Workstation LC/MS data acquisition software (Agilent Technologies, Santa Clara, CA, USA). The column employed in this study was a Waters BEH Acquity C18 (2.1 mm id × 100 mm, 1.7 μm)(Waters, Milford, MA, USA). The gradient eluent utilized consisted of acetonitrile (Mobile phase B) and 10 mM ammonium acetate solution, pH adjusted to 4.9 with acetic acid (Mobile phase A). Dataset for building QSRR models was obtained at column temperature 60°C and following gradient profile: Time = 0 min, %B = 5%; Time = 45 min, %B = 95% followed by 4 min equilibration. All other gradient profiles are specified in Table 4. All data were collected at column temperatures as specified in Table 4 and with an eluent flow rate of 0.4 mL/min. The injection volume was 2 μL and the UV detection was carried out at 254 nm.

### 3.2. Chemicals and Reagents

All standards used throughout the study were synthesized and characterized at Pfizer R&D UK Limited (Sandwich, UK). Standard solutions were initially prepared at 1 mg/mL concentration in diluent solution consisting of 50:50 (*v*/*v*) mixture of acetonitrile and water and stored in refrigerator. They were diluted 50-fold prior to injection with diluent. Acetonitrile (HPLC grade), ammonium acetate (LCMS grade) and acetic acid (Analytical grade) were purchased from Fisher Scientific (Loughborough, UK). Deionized water was prepared in house by MilliQ LC-Pak (Merck, Amsterdam, The Netherlands).

### 3.3. Software

AlvaDesc (Alvascience Srl, Lecco, Italy) software was used to calculate Dragon [13] descriptors (Formerly DragonX), Molecular Operating Environment (MOE, Chemical Computing Group Inc, Montreal, QC, Canada) software was used to calculate MOE descriptors and Molecular Discovery Software (Molecular Discovery, Borehamwood, UK) software was used to calculate VolSurf+3D descriptors [14]. Prior to descriptor calculation, 3D conformers were generated using Corina (Molecular Networks GmbH, Nürnberg, Germany and Altamira LLC, Columbus, OH, USA) followed by energy minimization using MMFF94 force field, embedded in MOE software.

WEKA [39] (version 3.8, Waikato, New Zealand) platform was used for feature selection and for the development and optimization of regression algorithms.

ACD/Labs LC Simulator (ACD/Labs, Toronto, ON, Canada) version 2019 was used to carry out two-dimensional resolution optimisation.

## 4. Conclusions

Chromatographic QSRR models were demonstrated to be useful for the prediction of retention times for hypothetical components with favourable accuracy. Likewise, the optimum resolution space was shown to be accurately represented when calculated using this approach. This was achieved by using a combination of Dragon, MOE and VolSurf+3D descriptors with a Support Vector Machine regression algorithm which outperformed all other tested conditions. An Evolutionary Search algorithm was used to reduce number of considered molecular descriptors from which the retention models were built. The retention times predicted from these models were used to build two-dimensional (gradient time versus temperature) resolution maps in order to identify optimal separation conditions. We found excellent agreement between the resolution of sample components obtained from a model built using experimental retention times with those from QSRR predicted retention times. These results indicate the usefulness of QSRR for the identification of optimal chromatographic conditions as well as for de-risking of existing methods for new/hypothetical components. It thus raises the prospect of an alternative approach to separation optimisation and de-risking that would not inherently rely on the availability of physical samples.

## Figures and Tables

**Table 1:** List of the 30 Most Frequently Selected Descriptors by Evolutionary Search.

Descriptor	Description	Descriptor	Description
CATS2D_03_DL	CATS2D Donor-Lipophilic at lag 03	LOGP_N-oct	Log Octanol/water
CATS2D_09_DA	CATS2D Donor-Acceptor at lag 09	CATS2D_09_NL	CATS2D Negative-Lipophilic at lag 09
F03[C-O]	Frequency of C-O at topological distance 3	GATS5s	Geary autocorrelation of lag 5 weighted by I-state
GATS6e	Geary autocorrelation of lag 6 weighted by Sanderson electronegativity	GATS6m	Geary autocorrelation of lag 6 weighted by mass
GATS7m	Geary autocorrelation of lag 7 weighted by mass	HATS4e	leverage-weighted autocorrelation of lag 4/weighted by Sanderson electronegativity
HATS5s	leverage-weighted autocorrelation of lag 5/weighted byI-state	Mor10p	signal 10/weighted by polarizability
AMW	average molecular weight	BLTA96	Verhaar Algae base-line toxicity from MLOGP (mmol/L)
Mor24p	signal 24/weighted by polarizability	N-075	R--N--R/R--N--X
nArCOOR	number of esters (aromatic)	NNRS	normalized number of ring systems
TDB07m	3D Topological distance-based descriptors—lag 7 weighted by mass	TDB08s	3D Topological distance-based descriptors—lag 8 weighted byI-state
a_acc	Number of hydrogen bond acceptor atoms	logS	Log of the aqueous solubility
PEOE_VSA_NEG	Total negative van der Waals surface area	PEOE_VSA+0	Sum of vi where qi is in the range of 0.00–0.05
SMR_VSA7	Sum of vi such that Ri > 0.56	ACACDO	H-bond acceptor and donor
L0LgS	Solubility profiling coefficient	L2LgS	Solubility profiling coefficient
pctFU4	Percent unionized species at pH 4	pctFU6	Percent unionized species at pH 6

**Table 2:** Regression Algorithms and their settings.

Algorithm	Settings
Support Vector Machine [36,37]	Normalized training data Polynomial Kernel
Gaussian Processes	Without hyperparameter tuning Normalized Polynomial Kernel
Multiple Linear Regression	M5 attribute selection method
Random Forest [38]	WEKA default Setting
Partial Least Squares (PLS)	Optimal Number of PLS factors determined using Leave One Out cross validation

**Table 3:** RMSE and R values for test sets at six screening conditions. See Table 4 for the experiment details.

	Experiment #1	Experiment #2	Experiment #3	Experiment #4	Experiment #5	Experiment #6
RMSE	0.4262	0.9981	0.3472	1.0133	0.4091	0.8401
R	0.9769	0.9763	0.9851	0.9792	0.9799	0.9874

**Table 4:** Screening sequence used to optimize the column temperature and gradient elution. See Materials and Methods for other conditions.

Experiment	Column Temperature (°C)	Gradient Profile ^a^
1	20	Time = 0 min, %B = 5%; Time = 15 min, %B = 95%
2	20	Time = 0 min, %B = 5%; Time = 45 min, %B = 95%
3	40	Time = 0 min, %B = 5%; Time = 15 min, %B = 95%
4	40	Time = 0 min, %B = 5%; Time = 45 min, %B = 95%
5	60	Time = 0 min, %B = 5%; Time = 15 min, %B = 95%
6	60	Time = 0 min, %B = 5%; Time = 45 min, %B = 95%

^a^ Followed by 4 min equilibration.

## Data Availability

Not applicable.

## Supplementary Materials

The following are available online at https://www.mdpi.com/article/10.3390/ijms22083848/s1, Table S1: QSRR predicted retention times from second screening, Table S2: Experimental retention times from second screening, Table S3: Selection of regression algorithm.

## Abbreviations

RP-LC	Reversed-Phase Liquid Chromatography
API	Active Pharmaceutical Ingredient
KPSS	Key Predictive Sample Set
QSRR	Quantitative Structure Retention Relationship
R	Correlation Coefficient
ES	Evolutionary Search
MLR	Multiple Linear Regression
RMSE	Root Mean Square Error
SVM	Support Vector Machine
GPR	Gaussian Processes Regression
RF	Random Forest
PLS	Partial Least Squares
RtModel_EXP_	Retention model built from experimental retention times
RtModel_QSRR_	Retention model built from QSRR predicted retention times
RC	Resolution Coefficient
Rs_limit_	Minimal satisfactory resolution between two components
Rs_i,j_	Actual chromatographic resolution between two components in the mixture
