# Supplementary material for: Structure Driven Prediction of Chromatographic Retention Times: Applications to Pharmaceutical Analysis
Source: Int J Mol Sci. 2021 Apr 8;22(8):3848. doi: 10.3390/ijms22083848 (PMC8068189; doi:10.3390/ijms22083848)
Supplement: Supplementary file 1 [file ijms-22-03848-s001.pdf]

International Journal of
Molecular Sciences

MDPI

Supplementary Materials

Structure Driven Prediction of Chromatographic Retention Times: Applications to Pharmaceutical Analysis

Roman Szucs [1,2,*], Roland Brown [1], Claudio Brunelli [1], James C. Heaton [1] and Jasna Hradski [2]

[1] Pfizer R&D UK Limited, Ramsgate Road, Sandwich, CT13 9NJ, United Kingdom; roman.szucs@pfizer.com (R.S.); roland.brown@pfizer.com (R.B.); claudio.brunelli@pfizer.com (C.B.); james.heaton@pfizer.com (J.C.H.)

[2] Department of Analytical Chemistry, Faculty of Natural Sciences, Comenius University in Bratislava, Mlynská Dolina CH2, Ilkovičova 6, SK-84215 Bratislava Slovakia; hradski1@uniba.sk (J.H.)

* Correspondence: roman.szucs@pfizer.com

Contents

Table S1. QSRR predicted retention times from second screening.
Table S2. Experimental retention times from second screening.
Table S3. Selection of regression algorithm.

Table S1. QSRR predicted retention times from second screening.

Compound ID	Retention time [min] Exp#1	Exp#2	Exp#3	Exp#4	Exp#5	Exp#6
Cmp-1	8.71	18.92	8.04	18.65	9.02	19.63[T2]
Cmp-2[b)]	8.71	20.29	9.04	20.85	9.02	20.80[T5]
Cmp-3[b)]	7.38	16.29	7.61	16.47	7.53	16.68[T5]
Cmp-4	6.24	10.15	6.37	10.80	6.49	10.70[T3]
Cmp-5	9.82	21.80	9.89	22.15	9.75	21.39[T7]
Cmp-6	6.37	16.11	6.43	15.99	6.55	15.51[T7]
Cmp-7	8.66	18.88	8.82	17.75	8.90	17.57[T8]
Cmp-8[a)]	7.86	18.82	8.04	18.68	8.15	18.66[T6]
Cmp-9	10.81	25.42	10.99	25.65	10.64	25.08[T4]
Cmp-10[b)]	11.06	25.76	11.08	26.25	10.79	25.74[T6]
Cmp-11	7.97	17.69	7.83	17.69	8.09	18.47[T1]
Cmp-12	6.72	14.60	6.55	14.19	6.36	13.16[T8]
Cmp-13	7.96	18.17	8.20	18.83	8.14	18.53[T8]
Cmp-14	7.48	16.29	7.36	16.87	7.45	16.27[T1]
Cmp-15[b)]	7.97	17.92	7.90	17.49	7.58	17.72[T5]
Cmp-16	8.45	18.76	8.59	17.48	8.60	18.58[T3]
Cmp-17	6.72	14.65	6.63	14.41	6.13	12.94[T2]
Cmp-18	6.77	15.19	6.72	15.59	6.71	14.87[T4]
Cmp-19[b)]	10.21	23.54	10.18	24.05	10.10	23.30[T2]
Cmp-20	7.30	15.27	6.86	14.58	7.10	14.45[T1]
Cmp-21	6.44	14.94	7.02	14.60	6.73	14.81[T6]
Cmp-22[b)]	6.91	15.01	6.89	14.81	6.75	15.07[T3]
Cmp-23	10.92	26.20	11.14	26.40	10.79	26.40[T7]
Cmp-24	10.95	24.90	11.25	25.47	11.01	24.70[T4]

[T1-T8] Indicates which training set / test set combination for building QSRR models compound belongs to.

[a)] Active Pharmaceutical Ingredient

[b)] Belongs to key predictive sample set

Table S2. Experimental retention times from second screening.

Compound ID	Retention time (min)					
	**Exp#1**	**Exp#2**	**Exp#3**	**Exp#4**	**Exp#5**	**Exp#6**
Cmp-1	7.74	17.47	8.05	18.03	8.17	18.33[T2]
Cmp-2[b)]	8.88	20.58	9.11	21.03	9.14	21.03[T5]
Cmp-3[b)]	7.33	16.49	7.51	16.72	7.61	16.79[T5]
Cmp-4	5.48	11.90	5.43	11.58	5.42	11.34[T3]
Cmp-5	9.80	22.64	9.62	23.82	9.68	22.18[T7]
Cmp-6	6.15	13.42	6.21	13.38	6.29	13.34[T7]
Cmp-7	8.91	18.93	9.12	18.78	9.16	18.34[T8]
Cmp-8[a)]	8.10	18.85	8.19	18.98	8.14	18.75[T6]
Cmp-9	10.98	25.09	11.09	25.78	10.95	25.61[T4]
Cmp-10[b)]	11.04	25.42	11.12	25.87	10.93	25.67[T6]
Cmp-11	7.91	18.10	8.08	18.34	8.14	18.32[T1]
Cmp-12	6.60	14.32	6.43	13.75	6.23	13.01[T8]
Cmp-13	7.52	17.15	7.64	17.23	7.71	17.22[T8]
Cmp-14	7.12	16.04	7.16	15.90	7.18	15.72[T1]
Cmp-15[b)]	8.10	18.56	7.91	17.94	7.61	17.08[T5]
Cmp-16	7.89	18.17	8.22	18.66	8.35	18.99[T3]
Cmp-17	6.44	13.87	6.15	12.94	5.83	11.94[T2]
Cmp-18	6.68	14.92	6.73	14.88	6.74	14.74[T4]
Cmp-19[b)]	10.40	23.83	10.51	24.29	10.36	24.10[T2]
Cmp-20	6.52	14.48	6.43	14.25	6.42	13.89[T1]
Cmp-21	6.40	13.51	7.22	13.54	6.41	13.34[T6]
Cmp-22[b)]	6.86	15.39	6.88	15.12	6.88	14.92[T3]
Cmp-23	11.92	27.73	11.87	28.01	11.59	27.59[T7]
Cmp-24	11.13	26.45	10.95	26.06	10.64	25.31[T4]

[T1-T8] Indicates which training set / test set combination for building QSRR models compound belongs to.

[a)] Active Pharmaceutical Ingredient

[b)] Belongs to key predictive sample set

Table S3. Selection of regression algorithm

(a) Support Vector Machine, (b) Gaussian Processes, (c) Multiple Linear Regression, (d) Random Forest, (e) Partial Least Squares

**Descriptors**		**Dragon**								**VolSurf+3D**							
**Training set**		**1**	**2**	**3**	**4**	**5**	**6**	**7**	**8**	**1**	**2**	**3**	**4**	**5**	**6**	**7**	**8**
(a)	RMSE	1.6584	1.6658	1.7665	2.0427	1.5736	1.588	1.5721	1.7428	1.5814	1.4009	1.4170	1.9217	1.3221	1.5715	1.2416	1.5520
	R	0.9673	0.9554	0.9703	0.9369	0.9725	0.9647	0.9778	0.9650	0.9882	0.9851	0.9779	0.9707	0.9863	0.9776	0.9891	0.9812
(b)	RMSE	1.4205	1.4788	1.3736	1.8152	1.4215	1.0301	1.4305	1.6584	1.4130	1.4360	1.9469	1.9923	1.6649	1.6589	1.4438	1.5239
	R	0.9725	0.9543	0.9740	0.9516	0.9679	0.9855	0.9661	0.9673	0.9665	0.9531	0.9150	0.9053	0.9495	0.9358	0.9678	0.9371
(c)	RMSE	1.2998	1.2418	1.2393	1.3774	1.2833	0.8505	1.1661	1.089	2.8519	3.4015	2.2541	4.6990	4.7040	6.6328	4.6274	3.6567
	R	0.9704	0.9658	0.9722	0.9704	0.9701	0.9841	0.9770	0.9785	0.8219	0.6904	0.8860	0.5737	0.3163	0.4315	0.0931	0.7176
(d)	RMSE	3.6412	3.4579	3.9424	3.896	3.6568	3.6828	3.7654	3.5971	2.2492	2.0436	2.2054	2.2480	2.1054	1.8418	2.3350	2.3101
	R	0.7530	0.7253	0.5888	0.6446	0.7884	0.7515	0.7089	0.6248	0.9327	0.9475	0.9451	0.9214	0.9492	0.9543	0.9378	0.9213
(e)	RMSE	2.5443	3.5571	4.9403	7.085	2.8096	6.4931	1.8485	3.2942	2.2450	1.5040	3.6301	2.5074	1.9649	1.9264	1.7360	1.9503
	R	0.8808	0.7874	0.7417	0.4257	0.8476	0.5282	0.9241	0.7756	0.8961	0.9435	0.6639	0.8412	0.9180	0.9184	0.9265	0.8965
**Descriptors**		**MOE**								**Dragon & VolSurf+3D**							
**Training set**		**1**	**2**	**3**	**4**	**5**	**6**	**7**	**8**	**1**	**2**	**3**	**4**	**5**	**6**	**7**	**8**
(a)	RMSE	1.9078	1.1811	1.3200	1.9778	1.1914	1.6002	1.1653	1.0252	1.2610	1.2870	1.3205	1.5626	1.8202	1.1620	1.2850	1.3282
	R	0.9335	0.9737	0.9716	0.9208	0.9815	0.9478	0.9786	0.9904	0.9799	0.9744	0.9756	0.9719	0.9569	0.9817	0.9817	0.9718
(b)	RMSE	3.2829	1.8448	1.8448	1.9988	1.8829	2.1857	1.5001	1.8355	1.1198	1.3997	1.1055	1.2241	1.3880	0.9925	0.9345	1.1905
	R	0.7669	0.9346	0.9346	0.9193	0.9330	0.9107	0.9652	0.9338	0.9764	0.9666	0.9789	0.9765	0.9604	0.9839	0.9831	0.9639
(c)	RMSE	6.5271	3.8470	3.8149	3.7128	5.3045	7.5378	3.9858	7.2548	1.1650	1.1873	0.9257	1.0834	1.9430	0.8783	1.0249	0.8778
	R	0.7334	0.8832	0.8662	0.8463	0.6921	0.7198	0.8929	0.2352	0.9725	0.9660	0.9830	0.9801	0.9287	0.9871	0.9759	0.9794
(d)	RMSE	2.6414	2.1795	2.3494	2.9233	2.7647	2.9066	2.6907	2.9207	2.8354	3.2253	2.9224	3.3266	3.2440	2.7972	2.9428	2.9209
	R	0.8767	0.9162	0.9097	0.8471	0.8804	0.8495	0.8872	0.8202	0.9113	0.8388	0.8893	0.8898	0.8616	0.9044	0.8776	0.9427
(e)	RMSE	1.8606	1.7642	1.6818	2.8430	2.6388	3.6273	1.6074	7.3690	2.4160	1.3216	1.3616	1.6998	4.7541	1.5540	1.4539	1.4646
	R	0.9277	0.9283	0.9374	0.7896	0.8535	0.6916	0.9370	0.4084	0.8939	0.9586	0.9574	0.9289	0.8079	0.9457	0.9494	0.9393

RMSE = Root Mean Square Error

R = Correlation Coefficient

Table S3. Continued

Descriptors		Dragon & MOE								VolSurf+3D & MOE							
**Training set**		**1**	**2**	**3**	**4**	**5**	**6**	**7**	**8**	**1**	**2**	**3**	**4**	**5**	**6**	**7**	**8**
(a)	RMSE	1.4262	1.2757	1.5206	1.7687	2.2811	1.3960	1.5419	1.6309	1.6212	0.8647	0.9396	1.3228	1.1210	1.0668	0.9905	1.0915
	R	0.9765	0.9757	0.9740	0.9581	0.9125	0.9745	0.9696	0.9664	0.9556	0.9884	0.9870	0.9701	0.9811	0.9815	0.9896	0.9795
(b)	RMSE	1.1259	1.0999	1.1816	1.3570	2.5260	1.2015	1.2100	1.1760	1.3744	1.0916	1.4141	1.1761	1.3807	1.3938	1.1312	1.2995
	R	0.9770	0.9729	0.9779	0.9701	0.8846	0.9731	0.9697	0.9696	0.9687	0.9745	0.9540	0.9684	0.9612	0.9564	0.9750	0.9532
(c)	RMSE	0.9823	0.9349	1.0880	1.2064	1.4710	1.0496	1.1803	1.0156	1.0742	0.9874	0.9199	1.2414	1.6013	1.7405	1.5456	1.3319
	R	0.9812	0.9793	0.9766	0.9702	0.9676	0.9778	0.9681	0.9720	0.9766	0.9768	0.9823	0.9640	0.9511	0.9490	0.9489	0.9520
(d)	RMSE	3.5149	3.1663	3.3953	3.5917	3.8944	3.2141	3.6650	3.3580	3.5278	3.0835	3.3572	3.4887	3.2732	3.2214	3.4319	3.4001
	R	0.7809	0.8028	0.7843	0.7880	0.6919	0.8941	0.6720	0.7390	0.9085	0.9299	0.9339	0.9314	0.9504	0.9341	0.9378	0.9213
(e)	RMSE	2.6829	2.4682	2.6154	1.8411	11.2558	1.6639	2.1227	1.6999	1.0469	1.9759	2.7317	1.5994	1.7688	3.1602	1.9598	1.2652
	R	0.8763	0.8864	0.8665	0.9172	0.6430	0.9373	0.9026	0.9249	0.9773	0.9050	0.8576	0.9391	0.9342	0.7841	0.9117	0.9531

Descriptors		Dragon & VolSurf+3D & MOE							
**Training set**		**1**	**2**	**3**	**4**	**5**	**6**	**7**	**8**
(a)	RMSE	0.9283	0.8429	1.0849	1.1695	0.9053	1.2082	0.8338	0.9697
	R	0.9739	0.9704	0.9804	0.9411	0.9838	0.9595	0.9870	0.9753
(b)	RMSE	0.9618	1.1913	1.1220	1.5570	1.1875	1.4656	1.1393	1.0062
	R	0.9883	0.9714	0.9796	0.9574	0.9758	0.9581	0.9729	0.9820
(c)	RMSE	0.9069	1.0744	1.0105	1.2837	1.0989	0.8678	1.0170	0.7408
	R	0.9845	0.9749	0.9798	0.9676	0.9775	0.9836	0.9782	0.9870
(d)	RMSE	3.3703	2.6197	3.4321	3.3555	3.3393	2.9612	3.1883	3.1900
	R	0.8469	0.9021	0.8331	0.8370	0.8621	0.8879	0.8391	0.8308
(e)	RMSE	1.9742	1.3900	2.2387	2.2214	1.6910	3.5238	1.7779	1.2862
	R	0.9207	0.9568	0.8682	0.6068	0.9417	0.7441	0.9330	0.9512

© 2021 by the authors. Submitted for possible open access publication under the terms and conditions of the Creative Commons Attribution (CC BY) license (http://creativecommons.org/licenses/by/4.0/).
